# The experiences of nursing students using virtual education during the COVID-19 pandemic

**DOI:** 10.25122/jml-2021-0315

**Published:** 2022-09

**Authors:** Seyedeh Narjes Mousavizadeh

**Affiliations:** 1 Department of Psychiatric Nursing and Management, School of Nursing and Midwifery, Shahid Beheshti University of Medical Sciences, Tehran, Iran

**Keywords:** educational management, virtual education, pandemic, Covid-19, nursing students

## Abstract

Due to the rapid spread of COVID-19, virtual education was proposed globally. This study aimed to examine the views and experiences of nursing students regarding quality, quantity, e-learning challenges, and solutions. This is a qualitative study using a purposive sampling method in which 42 nursing students were included. Data were collected through in-depth semi-structured face-to-face or telephone interviews and analyzed using content analysis. Concepts that were raised in the experience of nursing students were: "Incompatibility of educational processes", including ineffective teaching methods, limited interaction, limited feedback, low creativity, and educational injustice. "Loss of opportunities" including lack of clinical competence, concern for job opportunities, and lack of time management. "Imposed burnout", including forced labor and personal protection. "Personal helplessness", including lack of access to electronic facilities, struggles with the coronavirus, unemployment, and family conflicts. The enforcement of e-learning imposed restrictions on students with different conditions. Older students, those living in rural areas, students with work and family responsibilities, and people with limited electronic resources experienced challenges that require educational management based on challenges. Because e-learning goes beyond COVID-19 and given the continuing trend in e-learning in the coming years, it is necessary to address these challenges.

## INTRODUCTION

Given the rapid spread of COVID-19, which is highly contagious and can be fatal in severe cases, and the fact that no specific drugs are available for its definitive treatment, this is a significant threat to the lives and health of people in the community and has many effects on their emotional and coping responses [1]. On the other hand, there are still many doubts about the symptoms, transmission, and control of this disease. Hence, the spread of fear among people exposed to the virus is a fairly common phenomenon, and this leads to the greater focus of public health officials and the media on breaking the transmission chain through individual isolation, social distancing, and widespread closure of administrative and educational centers aimed at providing physical and psychological support to the society [2]. According to previous studies on infectious diseases and the announcement of the World Health Organization, home quarantine and avoidance of human gatherings can reduce the prevalence and pathogenicity of pathogenic microorganisms such as Covid-19 and thus, decrease the physical, psychological, social, and economic consequences of the widespread virus on infected people and people at risk [3]. In this regard, much attention was paid to virtual education as an alternative to face-to-face training in private and public educational institutions, and planning in this direction began [4, 5]. Today, e-learning is about to become one of the most crucial educational environments, as it can be an appropriate alternative to face-to-face training during an outbreak of infectious diseases [4] because the educator and the learner can communicate with each other through the tools and equipment provided by technology [6]. Research shows that virtual academic education is successful and efficient if the content is appropriately compiled and evaluated [7, 8]. Due to the changes caused by the outbreak of Covid-19, the importance of technology has become increasingly apparent, and almost every country in the world strengthened its online and virtual classroom learning platforms so that there is no interruption in education focusing on this issue. The most important tool in this platform is virtual class software. Also, another thought-provoking factor is the access and performance of students due to the immaturity of the method that needs to be studied and followed up [9].

The findings of a descriptive study on 400 students participating in e-learning centers of public universities in Tehran showed that these students, with full access to distance learning facilities in a given period, were satisfied with learning through this method and applying the e-learning approach. However, they did not have a positive attitude towards e-learning courses [10]. A study also reported that 94% of learners who completed distance learning courses believed that due to increased access and cost-effectiveness in e-learning, if sufficient educational support could be provided and learners' ability to make effective use of course-related technology would be considered, despite concerns about the distance learning approach, it can guarantee an increase in inclusive learning [11]. Of course, computer-assisted education also has its limitations, including that it cannot replace the teacher, the human and emotional interactions, and the face-to-face communication that takes place in a classroom [12]. Also, the supply and demand system of higher education does not yet have a precise understanding of virtual educational environments. Furthermore, it is not well acquainted with its capabilities and functions and does not yet know the basic information technology skills [13]. Although some educational institutions in recent years have offered complete distance learning courses or e-learning, not much evidence is provided about the evaluation results of these programs [14, 15].

The success of e-learning is not possible without considering people's views regarding this matter. However, students' attitudes and experiences at the end of a virtual course is a category that has received less attention in published studies. On the other hand, meaningful education is of great importance to nursing students due to the importance of acquiring special skills to provide care and acquire clinical competencies. Also, to ensure the acquisition of clinical skills in these students, there is a need for teaching and assessment objective methods, so in teaching skills, the traditional method still retains its special place and is used by teachers in colleges and educational institutions. Thus, the question in this research is which method is more effective in teaching sustainable skills. Also, can virtual education in medical sciences be considered a suitable alternative to traditional education? Accordingly, it is necessary to pay special attention to virtual education in medical sciences. Therefore, this qualitative study has comprehensively examined the attitudes and experiences of nursing students from participating in theoretical and practical training virtually during the Covid-19 pandemic crisis.

## MATERIAL AND METHODS

The present study comprehensively examines nursing students' experiences and the challenges of e-learning during the Covid-19 pandemic. Participants in this study, according to its nature and main purpose, were nursing students of the School of Nursing and Midwifery of Shahid Beheshti University of Medical Sciences who had been trained for at least one semester through e-learning and were included in the study by purposive sampling. Also, to achieve the greatest diversity, the researcher selected different participants in terms of gender, age, admission year, degree type, marital status, and employment status through theoretical sampling. Data collection was done through semi-structured and in-depth personal interviews in person or by telephone during the six months from November 2020 to April 2021. The time and manner of the interviews were determined according to the participants' conditions and desires, and the interviews were recorded with their consent. The duration of the interviews was 30–60 minutes, and each student was interviewed once or twice. The interviewer was one of the faculty teachers and was familiar with the students.

In the present study, after data collection, the conventional content analysis method was used [16]. The interviews were transcribed verbatim immediately or up to a maximum of 24 hours later and were reviewed several times to find a correct understanding of the whole case. Then, the semantic units were separated and identified in the text, and the text was coded by labeling each semantic unit. By comparing codes in terms of similarities and differences, similar codes were merged, and codes that represented a similar meaning and concept were placed in the same category. The classification and development of categories continued. With careful, deep reflection and comparison of the classes with one another, the themes were identified.

To ensure the scientific accuracy and validity of the data, the quality control method of the participants was used. The researcher also discussed, exchanged views, and reached consensus with expert professors in different stages of the study and also used the opinions of a colleague familiar with qualitative research to confirm the logical procedure of the findings.

## RESULTS

The current alert situation due to the COVID-19 pandemic led to an immediate change in the education of nursing students, as in other educational institutions, from face-to-face to distance education. This study aimed to explore the challenges and obstacles to this change, learning experiences, and expectations of students studying for undergraduate, graduate, and doctoral degrees in the School of Nursing and Midwifery of Shahid Beheshti University of Medical Sciences. This qualitative study was conducted in the second semester of virtual education in this school. Semi-structured interviews were conducted with 42 male and female students aged 18 to 49 years. Maximum variability in sampling was observed, and conventional content analysis was performed. Finally, four main themes were extracted:

1. Incompatibility of educational processes, including subclasses: ineffective teaching methods, limited interaction, limited feedback, low creativity, and educational injustice;
2. Loss of opportunities, including subclasses: clinical incompetence, job opportunities concern, and lack of time management;
3. Imposed burnout, including subclasses: forced labor and personal protection;
4. Personal helplessness, including subclasses: lack of access to electronic facilities, struggling with the coronavirus, unemployment, and family conflicts.

### The incompatibility of educational processes

This theme was mentioned in various aspects related to training by all interviewees. This uncertainty and indetermination were practically associated with ineffective teaching and assessment methods that cannot be resolved relatively quickly. The main challenges extracted in this category were: ineffective teaching methods, limited interaction, limited feedback, low creativity, and educational injustice.

Participants stated that various teaching methods are being used by different professors. In some of these methods, the quality and effectiveness are low, and even inconsistencies are observed.

*Only 2 out of 5 professors hold online classes; the rest upload files we have to understand* (Participant 14).

Since the efficiency and effectiveness of this type of teaching and learning depend entirely on the teaching approach of professors, empowering as many professors as possible in virtual teaching and evaluation methods is very important. Professors and students have experience with an education system that has never been 100% online and non-traditional, so they use different virtual education and evaluation strategies and have different views on e-learning, combination, or clinical education that confuse students.

*When there is no unity of procedure, students usually do not take classes and education seriously... Teachers need to spend more energy in online classes, use more creative methods to keep students active and convince them that these virtual classes are just as valuable as face-to-face classes and should be taken seriously* (Participant 6).

Participants noted that in most e-learning methods, a limited amount of learning is achieved. Interaction is necessary for an explanation, and a certain amount of pressure is required.

*The worst thing about e-learning is that when teachers only upload textbooks, no one forces you to read them... Later in the exam or homework, there are some hard-to-understand questions, and you cannot answer them* (Participant 25).

In addition, participants noted that one of the limitations of e-learning is that what relates to practical training is not learnable and requires more creative methods. The students identified this as a significant limitation and noted that practice is critical in nursing.

*Many things cannot be understood through a computer. Basic care skill labs are a good example. They can make videos and tell us how to do these procedures, or use simulation and advanced software..*. (Participant 5).

One of the main problems of e-learning is the relationship between teacher and student in the virtual and not real world. Therefore, since the students are not present in front of the teacher, if the teacher does not involve them in class, they might get out of touch with the group, and it will not be possible for the teacher to return them to the class circle. Additionally, cheating on tests and exams will be inevitable.

*As a result of virtual education and since our professors do not see us, everyone cheats easily… Usually, everyone achieves a high grade. When everyone gets a high grade effortlessly, it becomes tough for top students to have a high GPA; And it makes you feel your efforts are worthless* (Participant 9).

In this situation, professors have to look for new methods as classical teaching methods are often not applicable in this field. It seems that everything has to be rebuilt in educational centers to meet the new educational needs.

Participants believed that […] *teachers' indifference to the quality of education could lead to apparent educational injustice, the effect of which would remain on students' grades, scholarships, and other academic issues* (Participant 10).

### Loss of opportunities

The e-learning approach, according to many students, is a concern for the future because they will not be professionally prepared to enter the workplace as they do not receive an adequate virtual education. Among the issues raised in their experiences in this field were: clinical incompetence, concern for job opportunities, and lack of time management.

Clinical competence was a very significant practical component in nursing education and the most important aspect for students. The experiences found varied according to the groups of students. Depending on how close they were to the end of their training or whether they were in graduate school and needed skill-based training as nurses or health care professionals, they might find e-learning ineffective.

Some final year students stated that they would prefer not to graduate at the end of the semester, to complete all clinical education, and graduate later:

*Clinical education provides us with security in learning nursing care and health care services, which is why some of us prefer not to graduate in February* (Participant 22).

*In e-learning, clinical training was compulsorily limited. If this trend continues, our education will not be good* (Participant 35).

Some graduate students stated that the inability to receive specialized clinical education means losing job opportunities:

*If you do not receive clinical training, you will not learn specialized clinical work, so you will miss the opportunity to work in a specialized department from the beginning of your statutory service* (Participant 32).

Participants pondered time management and its impact on the future. This matter was crucial for final-year graduate students, who reported a great deal of wasted time. They could not go to clinical settings to do their dissertation and final projects and had only one option, to do them virtually with minimal facilities.

Some participants said that […] *even preparing for higher-level exams meant escaping their time-wasting condition and complete paralysis* (Participant 3).

### Imposed burnout

Students' entry into clinical settings was irregular and limited, and at the same time, despite the lack of personal protection equipment, it caused new stress for students and their families.

*In this critical condition, traveling from remote areas of the city or country to attend clinical settings specified by the university, due to transmission and infection risk on roads and in the hospital environment following all departments' involvement with the coronavirus, has worried students. As a result, our primary focus is on personal protection, and the emphasis on learning and performance is significantly reduced* (Participant 27).

### Personal helplessness

Not all students have access to electronic facilities. Lack of access to electronic facilities or high-speed internet at home and limited use of mobile internet in suburban or rural areas, network problems due to limited frequency bandwidth leading to connection problems, such as prolonged upload time and even data loss, microphone or camera failure, and even complete disconnection as a result of high data traffic and increased network users, may generate difficulties for many students.

*Some students refuse to attend online classes as they do not have adequate space and facilities at work or home, or they might be in a crowded and annoying environment. Some students can also not attend virtual or online classes due to a lack of smartphones or internet access at home* (Participant 18).

Some students, themselves, or their family members were infected, and some who were heads of households lost their jobs, and domestic violence has increased. Therefore, the students' mental state was very bad, so there was a need for more flexibility in education.

*Given the very different circumstances of different students, we need various ways of teaching, not a fixed way for everyone. Also, the use of different digital learning platforms and creating diversity and creativity in this field by the university for the benefit of all students with any facilities is an inevitable necessity* (Participant 31).

## DISCUSSION

Our findings showed that using new information and communication technologies in distance education could be one of the options for filling educational gaps in the absence of access to face-to-face education. However, how and to what extent this information and communication technology is used is an essential factor in the quality of education in various aspects of virtual education. The rapid shift to e-learning, while e-learning is not planned and people are not adapted, creates many problems, and our results present different ideas related to this topic. Firstly, empowering professors in virtual education and developing competencies in clinical education in different situations was essential from the point of view of all students. Ghaznavi (2018) also believes that the quantitative and qualitative expansion of teacher training should be commensurate with the growth and expansion of education. This means that the increase of faculty members and the expansion of specialized disciplines on the one hand and the specialization of faculty members in the required specialties on the other, as well as changes in educational programs, should be used for greater adaptation [17]. Studies show that in using new educational technologies such as e-learning, professors who have the knowledge and skills needed to achieve academic goals in these conditions are considered efficient [18, 19]. Secondly, increasing the use of digital learning platforms and creating diversity and creativity in this field using simulators and games leads to more learning in online education. Also, the simultaneous use of online education and feedback maintains the teacher-student interaction, which was identified in the findings of this study. Other studies found strategies such as appropriate exercises based on the scenario or immediate feedback, the integration of tutorial and participatory learning methods, and dynamic interaction in virtual education beneficial to facilitating e-learning [20, 21].

In order to maintain the learners' and teachers' robustness, in a situation where many options are not available to help those who become ill due to COVID-19, the education system has moved towards virtualization, and many activities have been done in this field. In the early stages, there was insufficient infrastructure for e-learning, and these limitations also seemed to affect student participation and motivation. Students missed some classes due to technical problems or had disappointing experiences attending classes. Initially, there was a challenge to previous experiences and knowledge about e-learning and the use of educational technologies. However, participants noted that the university is expected to ensure that online tools work flawlessly in later stages and train their instructors on how to proceed with their teaching duties to correct inconsistencies. Students are expected to easily acquire new routines, participate in online classes and seminars, simulation and advanced tools become more commonly used, and online training is facilitated because losing opportunities in any field can be detrimental to the stakeholders [22]. Other studies showed that since the beginning of the Covid-19 pandemic, universities worldwide have been taking rapid steps to ensure that students continue to learn through e-learning and secure student well-being, and the activities of each university in this field are unique [23, 24].

In the experiences of all participants, one of the most critical problems in online training was network problems, which happen due to limited frequency bandwidth and poor internet speed, constant disconnection, microphone or camera failure, and taking a long time to upload information. Even if the volume of data traffic is high and network users increase, complete disconnection occurs. Also, due to the impossibility or difficulty of accessing the internet, smartphone, or computer in the residence of different students, it is not possible to use e-learning equally for everyone. However, in other studies outside of Iran, these problems have been addressed only for students in remote and disadvantaged areas who have faced challenges such as access to technology equipment, poor internet connection, and challenging study environments, and universities are trying to provide solutions to these problems [25, 26].

Absolutely, in the e-learning approach, classical teaching methods are often not applicable. In this situation, educational centers and teachers are forced to look for new ways, making their workload even heavier. In this situation, preventing cheating on tests and exams is also one of the professors' concerns, which requires finding new and effective methods. Hence, following the lack of face-to-face communication in the real world and the impossibility of controlling students' participation, the possibility of cheating on tests and exams, unfair and superficial evaluations mentioned in the participants' experiences, the feeling of educational injustice, especially in the top students, are very bold. Other studies also suggest that special attention should be paid to student learning and assessment in online education. Adequate access to educational materials, articles, and e-books, as well as considering alternative assessment methods with the least risk of accumulation and the least error for students involved in online education, helps students to guide their learning efforts better. Professors can meet these needs by sharing valuable learning resources in the learning management system or in social media groups. Consequently, students are interested in emerging intelligent learning systems, which can provide a series of intelligent assessments for students [27].

Acquiring the component of clinical competency to maintain the labor market is a determining factor for people studying nursing, and the principal goal of these students is to have the opportunity of specialized clinical care. Academics have already discussed students' concerns about their future degrees and career advancement [28].

Our results support this idea in two ways: 1. The students showed that this attitude towards professional life is still valid in the new generations, with the remarkable fact that these students were very committed and eager to learn as much as possible. 2. The pandemic and its difficulties have reinforced their demands as nurses. Providing adequate personal protective equipment, retrieving planned and regular clinical training courses in the post-crisis period, utilizing clinical simulation to replace real environments, reducing pressure and stress, and increasing flexibility of the education system were among the students' expected essentials during this period. The results of other studies also discuss the return of nursing students to clinical settings and provide some recommendations [29]. Because these students are future nursing professionals, they must learn how to act in critical situations, so a balance between potential risk for students and the importance of clinical education must be considered [30].

Students prefer face-to-face learning to e-learning. In e-learning, their expectations of education are clearly shattered. With the possibility of some online courses being offered in conjunction with their traditional counterparts in the coming academic years, theoretical classes are preferred by students with family or professional responsibilities to be held exclusively online and recorded to be accessed at any time. Another aspect emphasized was that the traditional education system is a system they know and are accustomed to, and a complete change is not a profitable option for anyone. Face-to-face training is associated with communication, controls, and the assurance of proper evaluation, which can go beyond on-screen presentation. Other studies also suggest that the epidemic has forced a change of approach that academics still do not feel comfortable with, resulting in uncertainty and little security. This worries a large number of health academics [28, 30]. Therefore, it is necessary to work on compatibility with e-learning that considers the aspects mentioned above to reduce students' uncertainty, especially about assessments.

Nursing students considered the low quality of education and the importance of underlying factors as critical threats that must be addressed in online education. Use of various teaching-learning methods in theoretical and practical dimensions, accurate educational management, and quick executive measures to solve electronic problems, such as lending laptops with the internet for free to students in some universities, are urgent adaptations to this type of training method [24]. In the long run, providing sustainable solutions and calculated technology infrastructure is essential to the possibility of a continuing pandemic and distance education.

## CONCLUSION

The outbreak of coronavirus varies from one country to another. However, from an educational point of view, it is vital to identify emerging needs and priorities to continue educational activities in an appropriate environment to protect students' health. Implicitly providing non-discriminatory and equal conditions and education for all students, educational opportunities, supporting students with various vulnerabilities and using the facilities to reorganize educational processes by creating teams consisting of students, teaching staff, and experts outside of education is essential. Clinical education is essential for educating nursing students, and online education goes beyond continuing online theory classes. Therefore, learners should be able to use both offline and online platforms. In some cases, web-based training cannot meet users' needs. In addition to e-learning, it is necessary to organize face-to-face classes, which are mostly used in skill courses. Using blended learning, learners can interact with each other in the real world while interacting with tools on the web, and given the uncertain future of COVID-19, officials need to work on this for the next school year.

## References

[1] Wang C, Pan R, Wan X, Tan Y (2020). Immediate Psychological Responses and Associated Factors during the Initial Stage of the 2019 Coronavirus Disease (COVID-19) Epidemic among the General Population in China. Int J Environ Res Public Health.

[2] Wang C, Pan R, Wan X, Tan Y (2020). A longitudinal study on the mental health of general population during the COVID-19 epidemic in China. Brain Behav Immun.

[3] The Lancet Psychiatry (2020). Isolation and inclusion. Lancet Psychiatry.

[4] UNESCO COVID-19 Educational disruption and response 2020. https://en.unesco.org/themes/educationemergencies/coronavirus-school-closures.

[5] Strielkowski W (2020). COVID-19 Pandemic and the Digital Revolution in Academia and Higher Education. Preprints.

[6] Kian M (2014). Challenges of virtual education: A report of what are not learned. Interdisciplinary Journal of Virtual Lerning in Medical Sciences.

[7] Bali S, Liu M (2018). Students' perceptions toward online learning and face-to-face learning courses. Journal of Physics: Conference Series.

[8] Platt CA, Raile A, Yu N (2014). Virtually the same? Student perceptions of the equivalence of online classes vs. face-to-face classes. Merlot Journal of Online Learning and Teaching.

[9] Pandya K, Gor K (2018). Knowledge management: A success key for higher education. Fed Uni Journal of Higher Education.

[10] Jahanian R, Etebar Sh (2012). The Evaluation of virtual education in view point virtual e-learning centers in universities of Tehran from students. Information and Communication Technology in Educational Sciences.

[11] Nikoonezhad S, Zamani BE (2014). Comparison of interaction and social presence of virtual and non-virtual students in terms of demographic factors and academic achievement. Applied Sociology.

[12] Zeraati M, Zakipour M, Aghabararian N (2015). Comparison of Lecture and Network-Based Educational Methods on Improving the Academic Performance of Students; Mazandaran University of Medical Sciences. Bimonthly of Education Strategies in Medical Sciences.

[13] Kebritchi M, Lipschuetz A, Santiague L (2017). Issues and challenges for teaching successful online courses in higher education. Journal of Educational Technology Systems.

[14] Aldhafeeri FM, Badrul HKh (2016). Teachers' and Students' Views on E-Learning Readiness in Kuwait's Secondary Public Schools. Journal of Educational Technology Systems.

[15] Ganbari S, Rezghi Shirsavar H, Ziaee MS, Moslem M (2019). Evaluating the Effectiveness of Virtual Education on Health Care Management Students. Journal of Healthcare and Management.

[16] Strauss A, Corbin J (2015). Basics of Qualitative Research: Techniques and Procedures for Developing Grounded Theory.

[17] Ghaznavi MR, Najjari M, Rahimi AM (2018). Investigating the role of new educational technologies in the teaching efficiency of teachers. New National Conference on Psychology, with emphasis on its applications in work and life. Isfahan.

[18] Nosrati S, Jangi Zehi HR (2019). Investigating the role of new technology (internet) in achieving educational quality 11^th^ International Conference on Psychology and Social Sciences, Tehran, Mehr Ishraq Conference. https://www.civilica.com/Paper-RAFCON11-RAFCON11_226.html.

[19] Kouhestani Nejad Tari A, Abazari Z, Mir Hosseini Z (2018). Teacher technology literacy in the document of the national curriculum of education in the field of training and learning of work and technology. Journal of Educational Technology.

[20] Yan L, Whitelock-Wainwright A, Guan Q, Wen G (2021). Students' experience of online learning during the COVID-19 pandemic: A province-wide survey study. Br J Educ Technol.

[21] Azizifar MJ, Mohammadian A, Safari I (2016). Provide an e-learning model based on strategic and architectural perspectives. 2 ND International Conference on Management & Industrial Engineering, Istanbul, Turkey.

[22] Mousavizadeh SN, Banazadeh Z (2020). Loss of Time in the Treatment Adherence Process: A Qualitative Study in a Sample of Iranian People with Diabetes. Journal of Medicine and Life.

[23] Dhawan Sh (2020). Online Learning: A Panacea in the Time of COVID-19 Crisis. Journal of Educational Technology Systems.

[24] Basilaia G, Dgebuadze M, Kantaria M, Chokhonelidze G (2020). Replacing the classic learning form at universities as an immediate response to the COVID-19 virus infection in Georgia. International Journal for Research in Applied Science & Engineering Technology.

[25] Alsoud AR, Harasis AA (2021). The Impact of COVID-19 Pandemic on Student's E-Learning Experience in Jordan. Journal of Theoretical and Applied Electronic Commerce Research.

[26] Carey K Is everybody ready for the big migration to online college? Actually, no. The New York Times.

[27] Butnaru GL, Nit V, Anichiti A, Brînză G (2021). The Effectiveness of Online Education during Covid 19 Pandemic-A Comparative Analysis between the Perceptions of Academic Students and High School Students from Romania. Sustainability.

[28] Martin A (2020). How to optimize online learning in the age of coronavirus (COVID-19): A 5-point guide for educators. Journal of Educational Technology Systems.

[29] Zalat MM, Hamed MS, Bolbol SA (2021). The experiences, challenges, and acceptance of elearning as a tool for teaching during the COVID-19 pandemic among university medical staff. PLoS ONE.

[30] Kaur N, Dwivedi D, Arora J, Gandhi A (2020). Study of the effectiveness of e-learning to conventional teaching in medical undergraduates amid COVID-19 pandemic. Natl J Physiol Pharm Pharmacol.

